# Hemothorax After a Renal Biopsy With Ablation, a Rare Complication: A Case Report and Review of the Literature

**DOI:** 10.7759/cureus.12439

**Published:** 2021-01-03

**Authors:** Pahnwat T Taweesedt, Humayun Anjum, Rahul Dadhwal, Salim Surani

**Affiliations:** 1 Pulmonary Medicine, Corpus Christi Medical Center, Corpus Christi, USA; 2 Internal Medicine, University of North Texas, Dallas, USA

**Keywords:** complication, hemothorax, kidney biopsy, renal biopsy

## Abstract

Renal biopsy is an important diagnostic test which is used to extract kidney tissue with the help of a biopsy needle. It is frequently performed under ultrasonography or CT guidance. As with every other procedure, renal biopsy also carries some risks. Common complications of renal biopsy are damage to adjacent organs. Hemothorax is an exceedingly rare complication of renal biopsy. We report a case of a middle-aged female who developed a left-sided hemothorax after undergoing percutaneous renal biopsy with ablation and conducted a literature review of this rare complication.

## Introduction

Renal biopsy is an essential tool to establish the diagnosis and evaluate the treatment for patients with kidney diseases such as nephrotic syndrome, glomerulonephritis, unexplained acute kidney injury, systemic disease with renal involvement, renal transplant rejection, and renal mass. Renal biopsy can be done by percutaneous or nonpercutaneous approaches including open, laparoscopic, and transjugular kidney biopsy [1-2]. Percutaneous renal biopsy is a preferred method in most patients as it is less invasive compared to nonpercutaneous renal biopsy. Percutaneous renal biopsy can obtain renal tissue using a biopsy needle under ultrasonography, CT, or MRI-guidance to penetrate the renal capsule [3]. In a recent large meta-analysis of percutaneous native kidney biopsy complications, the common complications were perinephric hematoma (11%), pain (4.3%), and macroscopic hematuria (3.5%) [4]. Albeit rare, mortality can occur in 0.06% of these patients [4].

Hemothorax is referred to as the blood in pleural space. It is often caused by a blunt or penetrating thoracic injury. Hemothorax was reported in 0.2% and 0.1% of percutaneous lung and liver biopsies, respectively [5-6]. To date, there have been only a few case reports of hemothorax due to renal biopsy [7-9]. We report a rare case of a middle-aged female who developed left hemothorax after percutaneous renal biopsy with ablation and reviewed literature of the previously reported cases with this particular complication.

## Case presentation

A 47-year-old woman with chronic kidney disease stage IIIa, endometrial cancer (status post hysterectomy and bilateral oophorectomy six years ago), bilateral renal stone, and left renal tumor with suspected malignancy presented to the ED with one day of shortness of breath, left side pressure, and pleuritic chest pain radiating to the left shoulder. One day before the ED visit, she underwent a biopsy of the left renal superior pole tumor followed by microwave ablation under CT guidance without immediate post-procedure complication. Post-biopsy images showed some perinephric fluid/blood anteriorly, which did not increase significantly throughout the procedure. She had minimal dizziness but denied fever, palpitation, cough, hemoptysis, hematuria, hematochezia, abdominal pain, or similar symptoms in the past. She did not take antiplatelet medication or any other anticoagulation before the procedure.

**Figure 1:**
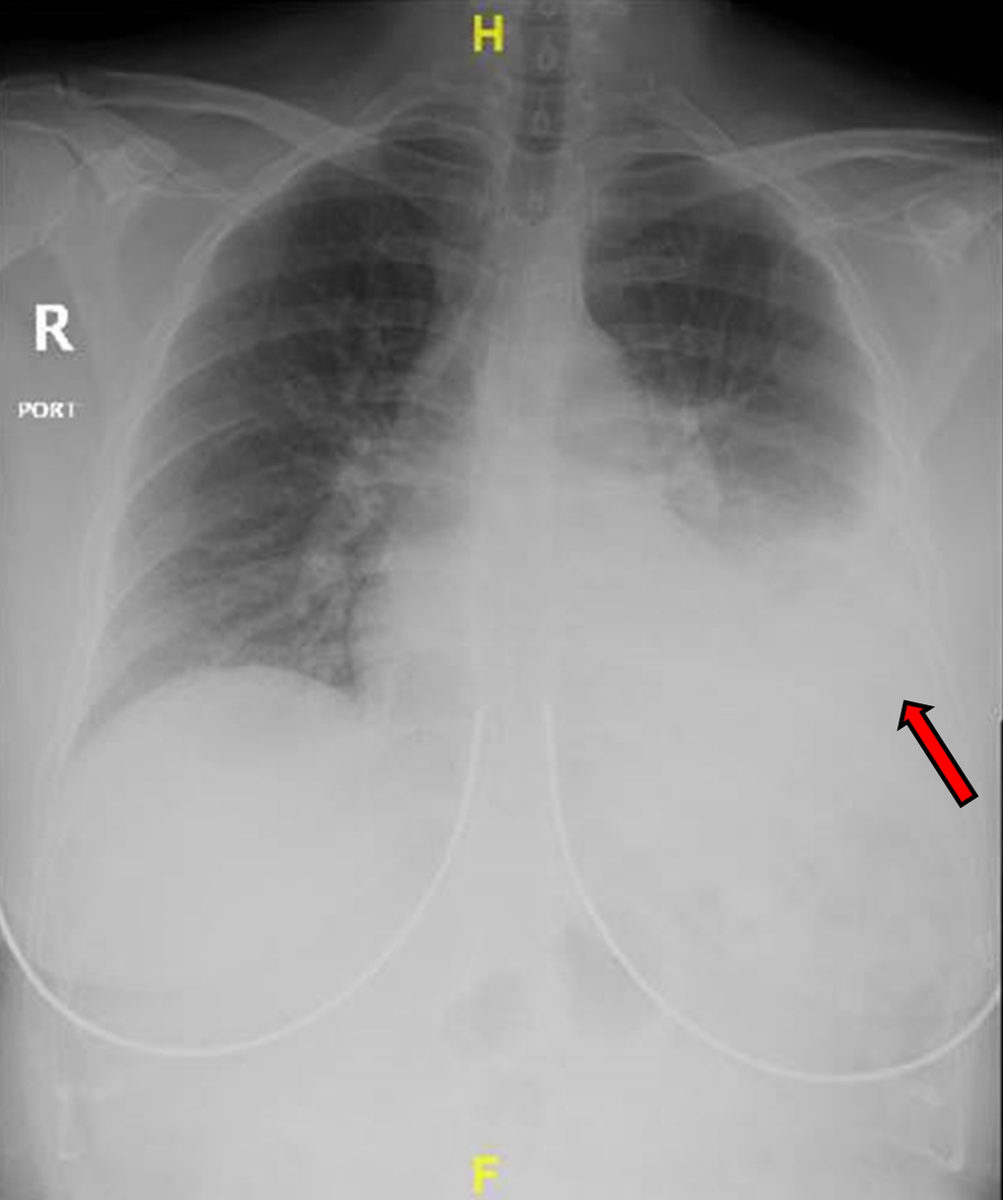
Chest X-ray showing moderate left pleural effusion (red arrow).

**Figure 2:**
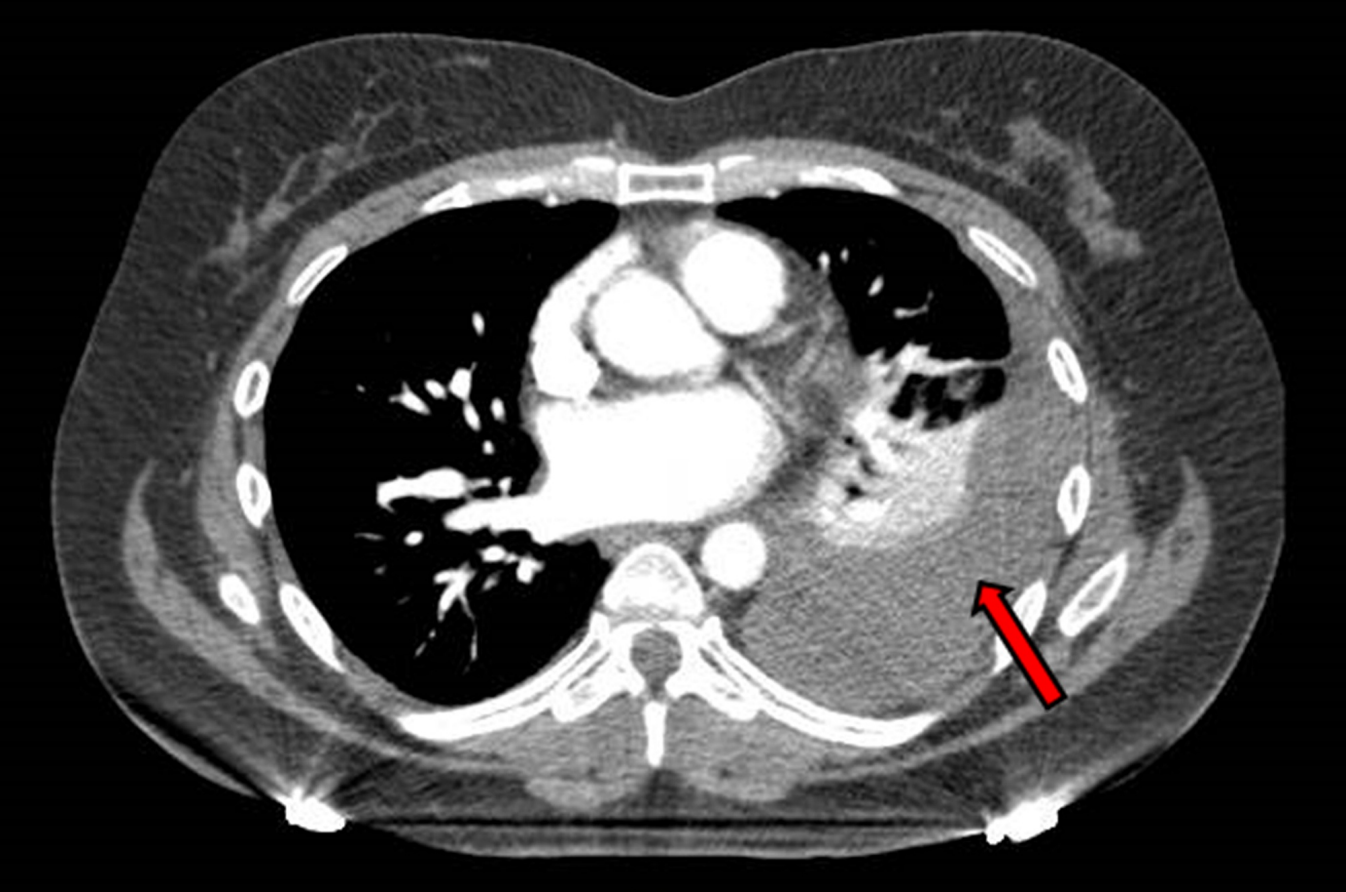
CT of the chest showing moderate to large left pleural effusion with left lung atelectasis (red arrow).

She was afebrile and had a blood pressure of 154/70 mmHg, pulse rate of 88/min, and respiratory rate of 18/min. There was no desaturation on room air. Physical examination was remarkable for decreased breath sounds in the left lower lung field. Labs showed white blood cell count of 6,400/µL, neutrophil of 60%, hemoglobin of 9.8 g/dL (baseline hemoglobin 13.5 g/dL one day prior), mean corpuscular volume (MCV) of 93.2 FL, platelet of 239,000/µL, prothrombin time (PT) of 10.5 s, INR of 0.93, and partial thromboplastin time (PTT) of 27.8 s. Troponin was negative. Electrolytes were within normal limits, and renal function was at baseline. Electrocardiogram showed no significant ST-T change. Chest X-ray showed a moderate-sized left pleural effusion (Figure 1). CT scan of the chest with angiography confirmed moderate to large left pleural effusion without evidence of pulmonary embolism or intrathoracic mass lesion (Figure 2).

Thoracentesis was done. Hemothorax was diagnosed as bloody fluid 500 mL was obtained. A pigtail catheter was subsequently placed. Pleural fluid showed red blood cells (RBC) of 71,000/mm3, white blood cell count of 3,969/mm3, total protein of 5 g/dL (serum total protein 6.6 g/dL), and lactate dehydrogenase (LDH) of 293 U/L (serum LDH 155 U/L). No organisms grew in pleural fluid culture. Serum hemoglobin was monitored and noted to drop to 8.9 g/dL without hemodynamic change. After that, hemoglobin remained stable and no blood transfusions were required. CT of the abdomen and pelvis revealed atrophic change at the upper pole of the left kidney without free fluid or inflammatory change in the peritoneal cavity. There was a significant decrease in left pleural fluid on the repeat chest X-ray. Pigtail catheter was removed after five days as the output was less than 100 mL in 24 h. Hemoglobin upon discharge was 10 g/dL. The patient was discharged home and did not have respiratory complaints on one week follow up. Renal biopsy and the pleural fluid did not show malignant cells.

## Discussion

Renal biopsy may be performed before or during the same session as ablation for a small renal mass. The latter method is considered less risky. Nonetheless, the combined renal biopsy-ablation procedure may result in a higher rate of benign tumor ablation and nondiagnostic pathology results [10]. Our patient underwent renal biopsy and ablation in the same visit for her small left renal mass. In addition to renal biopsy complications, a renal biopsy with ablation can also cause thermal injury and post-ablation syndrome which include pain, low-grade fever, nausea, vomiting, malaise, and myalgias [11]. Hemothorax is very rare for both renal biopsy alone and renal biopsy with ablation. In a retrospective study of 164 cryoablations and 84 radiofrequency ablations by Hegarty et al., only one patient developed hemothorax in cryoablation and none in the radiofrequency ablation group [12]. Turna et al. found one subject with hemothorax among 216 percutaneous cryoablations [13].

Hemothorax can be due to bleeding from pleura or extrapleural structures such as chest wall tissue, intercostal, and internal mammary vessels. Moreover, bleeding from intrathoracic structures such as the diaphragm, heart, mediastinum, lung parenchyma, or metastatic lesion can also cause hemothorax. Posteriorly, phrenic and intercostal vessels are located along the lower border of the rib behind the kidney. Also, the diaphragm and parietal pleura are attached to the lower portion of the 12th rib. Insertion of the probe may cause injury to bleeding into the probe tract and pleural space [7]. In our case, hemothorax was likely caused by intercostal vessel injury during the needle placement as the upper portion of the kidney is slightly covered by the 11th and 12th rib cage.

Among patients who had major complications postrenal biopsy (severe bleeding requiring transfusion or invasive procedure, septicemia, acute renal obstruction, acute kidney injury, or death), 38%, 67%, 80%, and 91% of these patients had cumulative timing of complications within 4, 8, 12, and 24 hours, respectively [14]. Hemothorax can lead to hemodynamic and respiratory compromise. Dyspnea and chest pain are common complaints similar to our patient’s presentation. Respiratory symptoms in our patient improved significantly after the drainage. Chest X-ray is the primary investigation for hemothorax. Nonetheless, the CT chest demonstrates better localization and quantification of blood in pleural space. Pleural fluid hematocrit more than 50% of serum hematocrit is considered a hemothorax. Unfortunately, pleural fluid hematocrit could not be done for our patient as it was not available in the hospital laboratory. Retained hemothorax with bacterial contamination can lead to empyema, sepsis, and septic shock. Without drainage, organized hemothorax subsequently begins after the first week, followed by the fibrous scar, entrapped lung, and decreased pulmonary function [15]. The pigtail catheter was placed for the treatment of hemothorax in our patient, which helped to drain the blood out.

**Figure 3:**
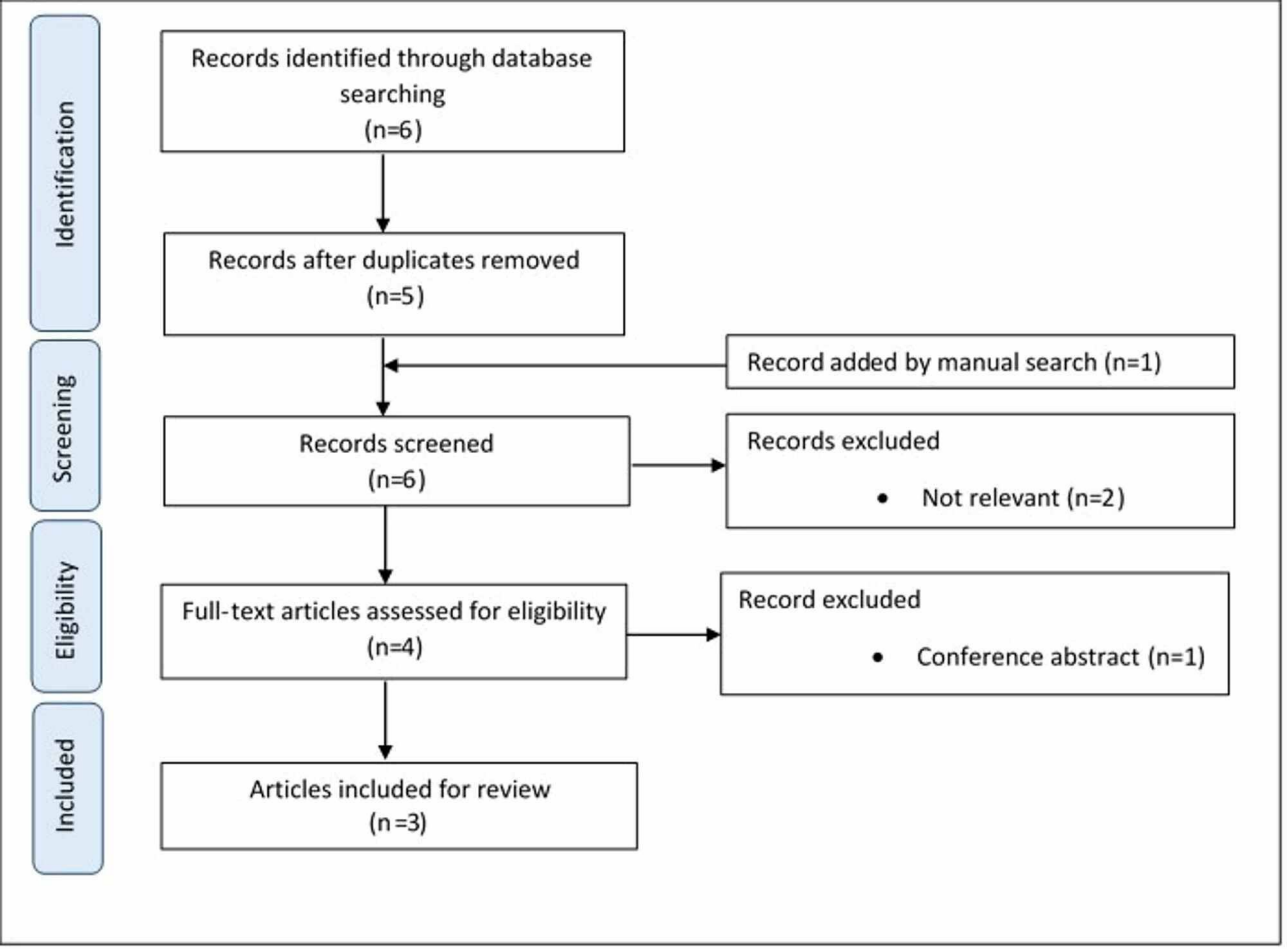
Flow diagram for article selection according to PRISMA 2009 guideline.

We conducted a literature review of renal biopsy with hemothorax complication. We searched Pubmed and EMBASE from inception to December 2020 using the keywords “renal biopsy”, “kidney biopsy,” and “hemothorax” (Figure 3). A total of three prior reported cases were found (Table 1). All patients including ours were female with a mean age of 49.8 years old. Based on previous studies, the female gender is one of the common risk factors for post-renal biopsy bleeding apart from advanced age, high blood pressure, high serum creatinine, and bleeding diatheses [16-17]. Flank pain, chest pain, and dyspnea were presenting symptoms. Hemothorax onset after renal biopsy ranged from 2.5 hours to three days. Location of renal biopsy varies from upper pole, lower poles, and mid-portion of the kidney. Three patients developed hemothorax on the left side. The severity of hemothorax ranged from moderate to massive hemothorax. All four cases received pleural fluid drainage (two cases with chest tubes and two cases with pigtail catheters). Three patients received a blood transfusion. All patients were discharged alive.

**Table 1:** Summary of case reports of hemothorax complication of renal biopsy. AF, atrial fibrillation; AKI, acute kidney injury; bx, biopsy; CHF, congestive heart failure; CKD, chronic kidney disease; COPD, chronic obstructive pulmonary disease; F, female; HTN, hypertension; M, male; NA, not applicable; TTP, thrombotic thrombocytopenic purpura; US, ultrasonography

First author (year)	Gender, age (years)	Signs and symptoms	Hemothorax side, severity	Renal biopsy indication, location	Renal bx type	Bx location	Onset after bx	Co-morbidity	Treatment/outcome
Bissler JJ (1991) [7]	F, 17	Right flank pain, tachypnea	Right, moderate (600 ml)	Lupus nephritis disease activity evaluation	Percutaneous renal bx with fluoroscopy	Lower pole	2 ½ hours	Lupus nephritis, obesity	Chest tube 2 days, blood transfusion/survive
Lo YH (2017) [8]	F, 48	Left-flank pain, shock	Left, massive With large perinephric hematoma	AKI	Percutaneous renal bx with US	NA	2 days	HTN, TTP	Pigtail catheter, blood transfusion/survive
Romero FR (2007) [9]	F, 87	Dyspnea, chest pain	Left, large	Renal mass	Percutaneous renal bx with CT and cryoablation	Mid-portion	3 days	COPD, HTN, CHF, AF	Chest tube, blood transfusion/survive

## Conclusions

Hemothorax is a rare consequence of renal biopsy which can happen in a few hours and up to 3 days after the biopsy. Awareness of this complication is essential to help with early diagnosis and patient care. Real-time imaging guided percutaneous renal biopsy may help prevent this complication. The treatment of choice for a hemothorax is tube thoracostomy drainage which is recommended to avoid future complications.
